# #MEDToo – sexual harassment in medical education: perceptions and coping strategies of medical students in Germany, a qualitative study

**DOI:** 10.1186/s12909-026-09090-1

**Published:** 2026-04-02

**Authors:** Sabine Drossard, Iris Warnken, Marco Kuchenbaur, Anja Härtl, Inga Hege

**Affiliations:** 1 Department of General, Visceral, Transplant, Vascular and Pediatric Surgery, University Hospital Würzburg, Oberdürrbacher Str. 6, Würzburg, 97080 Germany; 2 Medical Didactics and Education Research, DEMEDA, Faculty of Medicine, University of Augsburg, Augsburg, Germany; 3 Department of Hygiene and Environmental Medicine, University Hospital Augsburg, Augsburg, Germany; 4 Personal and Interpersonal Development in Health Care Education, Witten/Herdecke University, Witten, Germany; 5 Institute of Research in Health Sciences Education & Faculty of Health Sciences, Medizinische Hochschule Brandenburg Theodor Fontane, Neuruppin, Germany

**Keywords:** Sexual Harassment, Gender-Based Discrimination, Professional Identity Formation, Medical Education, Medical Studies, Students, Final Year, Undergraduate Education, Workplace Violence, Interview Study

## Abstract

**Introduction:**

Sexual harassment is a form of power abuse prevalent in healthcare, with medical students experiencing it frequently, especially in practical training. A high proportion of medical students in Germany experience harassment or discrimination during their education, yet detailed data on their perceptions and coping strategies in the clinical environment are lacking.

**Aim:**

This study aims to analyze the experiences of final-year medical students in Germany with sexual harassment, identify factors that hinder or support coping, and offer recommendations for preventive measures and support services.

**Methods:**

We conducted semi-structured, guideline-based individual interviews with medical students in their final year of medical training at the University Hospital Augsburg (UKA) who reported a history of sexual harassment during their studies. We analyzed the data using Kuckartz’s qualitative content analysis method.

**Results:**

We conducted twelve interviews with ten female and two male medical students. Our analysis revealed five interrelated themes illustrating how experiences of gender-based discrimination and sexual harassment intersect with processes of professional identity formation within hierarchical medical training environments. First, participants described a spectrum of gendered boundary violations occurring in both educational and clinical relationships. These experiences were shaped by the specific relational context and involved supervisors within hierarchical training structures and patients within therapeutic encounters. Second, such incidents were closely intertwined with students’ emerging professional identities, often generating uncertainty in interpretation and tension between maintaining professional conduct and protecting personal boundaries. Third, rigid hierarchies and cultural normalization within medical training environments reinforced silence and limited students’ willingness to challenge inappropriate behavior. Consequently, students often adopted adaptive strategies characterized by restraint, minimization, or strategic silence. Finally, participants articulated the need for institutional structures, cultural change and practical skills to enable them to set professional boundaries with confidence.

**Conclusion:**

Students’ narratives reflect a dynamic interplay between gendered boundary violations, role insecurity, hierarchical dependency, constrained agency, and perceived gaps in institutional support. Sustainable prevention of SH in medical education requires both structural reforms and educational programs to enhance individual competencies.

**Supplementary Information:**

The online version contains supplementary material available at 10.1186/s12909-026-09090-1.

## Introduction

Sexual harassment (SH), as defined by the German General Act on Equal Treatment *(Allgemeines Gleichbehandlungsgesetz* – AGG), includes any unwanted, boundary-violating, or degrading sexualized behavior and may be physical, verbal, or non-verbal in nature [1]. It constitutes a specific form of power abuse, particularly in the workplace [1]. Closely related, gender-based discrimination (GBD) refers to the systematic disadvantage or devaluation of individuals based on their gender [1]. While SH is often conceptualized as a specific form of GBD, not all gender discrimination involves sexualized behavior. Both phenomena are rooted in structural gender inequalities and power asymmetries, yet they differ in manifestation and legal framing.

SH is a widespread workplace issue. According to the 2022 report of the German Federal Anti-Discrimination Agency, 9% of employees (13% of women and 5% of men) experienced SH within three years [2]. Healthcare workers are particularly affected. While misconduct by patients is often normalized as a “professional risk” [2], harassment by co-workers and supervisors within hierarchical workplace structures poses additional challenges [3].

In medical education, international studies report that up to one-third of students experience SH and over half face GBD [4, 5]. Similar rates have been observed in Germany, where approximately 50% of medical students experienced harassment or discrimination [6–9]. National data further suggest increasing prevalence during clinical training, with 67% of final-year students (75% of women) reporting at least one incident [10]. Among physicians, 74.2% of women and 51.2% of men reported workplace harassment in a 2022 multi-center study [11].

Previous research demonstrates that SH in medical school is associated with substantial adverse mental health and academic consequences. Students who experienced SH report higher levels of depression and post-traumatic stress symptoms, greater stress, suicidal ideation, substance use, and burnout, as well as increased likelihood of seeking mental health care [12–14]. Moreover, SH is linked to reduced academic engagement, lower institutional and career satisfaction, and perceptions of limited faculty support [13, 14].

Despite its high prevalence, evidence-based guidance for addressing SH in medical education remains scarce. This qualitative study explores how final-year medical students experience, interpret, and respond to harassment during their training. We aim to identify types of harassment, their impact, and the personal and structural factors shaping students’ reactions and perceptions of institutional support. Based on these insights, we propose recommendations for prevention and support within medical faculties and teaching hospitals.

## Methods

We conducted this qualitative study with final year medical students at the University Hospital Augsburg (UKA), using semi-structured, guideline-based interviews to explore their experiences with SH during medical training.

### Sample description and recruitment

We selected participants using purposeful sampling [15], targeting final-year medical students at UKA who had experienced SH during clinical training. As UKA hosted students from medical schools across Germany, this setting enabled maximum variation sampling [16]. We recruited participants via email, posters at the hospital, and direct contact during orientation events; interviews were scheduled after initial contact. The final sample size was guided by data saturation, defined as the point at which no new codes or themes emerged from successive interviews.

### Development of the interview guide

SD developed the semi-structured interview guide based on relevant literature on SH in workplace and healthcare settings [3, 4, 6, 7, 13, 17–24], and theories of professional identity formation in medical students [25, 26]. The design followed Helfferich’s 4-step SPSS method [27], ensuring theoretical grounding and coherence, and incorporated Przyborski and Wohlrab-Saar’s principles of “Openness,” “Specificity,” and “Contextualization and Relevance” [28]. A pilot interview tested clarity and structure; its data were excluded from analysis. A brief demographic questionnaire captured participants’ age, gender, previous work experience, and cultural background. The final interview guide and questionnaire are available in Supplement 1.

### Interview process

SD, a female pediatric surgeon, who had no supervisory relationship with participants, conducted the interviews in a neutral setting outside the clinical environment. Participants received verbal and written information about the study and provided informed consent. After completing a demographic questionnaire, they received a written definition of SH (based on the General Equal Treatment Act, AGG) with illustrative examples (Supplement 1). Interviews began with the open question “Have you ever experienced sexual harassment during your medical studies? Can you tell me about it?” to stimulate storytelling. Follow-up questions were used for clarification or elaboration as needed (Supplement 1). If participants answered “no,” the interview was concluded.

### Data analysis

We audio-recorded the interviews and pseudonymized and transcribed verbatim by an external service provider in accordance with data protection regulations. We analyzed the transcripts using MAXQDA Plus 24 (VERBI GmbH, Berlin), following Kuckartz’s structured content analysis approach [29, 30]. Categories were developed deductively based on literature and inductively from the data. For instance, emotions related to SH were derived from existing research on interpersonal violence and refined through textual analysis. SD conducted the initial coding, defining main and subcategories, which were reviewed and discussed with AH, IH, IW, and MK to ensure methodological rigor. IH, IW, and MK independently coded one interview excerpt to establish consensus and enhance intercoder reliability [31]. The final coding framework was validated across the entire dataset. The study adhered to COREQ-32 guidelines [32] and established quality criteria for qualitative research, including transparency, intersubjectivity, and comprehensiveness [33].

## Results

We conducted twelve interviews between August and November 2023 with ten female and two male final-year medical students. Saturation was reached after ten interviews, as no new categories emerged. Two additional interviews were conducted to confirm thematic completeness and ensure representational depth. Participants had a mean age of 25.6 years (median = 25; range: 24–27; SD = 1.0). All participants were German, four of them reported prior medical experience. Interviews averaged 16.0 min (mean = 14.6; range = 10.4–26.4; SD = 4.41). Those with male participants were shorter (mean = 11.0; SD = 0.79) than with female participants (mean = 17.1; SD = 4.10). All female students reported personal experiences of SH, while the two male students described witnessing but not experiencing it themselves. Our analysis revealed five interconnected dimensions:

### Theme 1: gendered and sexualized boundary violations

Students described a wide spectrum of SH and GBD occurring across clinical and educational settings (Table 1). These ranged from subtle forms of gendered devaluation—such as diminutive forms of address, condescending remarks, stereotypical assumptions about career aspirations or motherhood, or being mistaken for nurses—to structural disadvantages, including reduced access to teaching opportunities or gendered expectations regarding career choices. Students also reported the reinforcement of gender stereotypes through jokes, sexist remarks, and comments about women’s roles, as well as objectifying comments about appearance. In addition, participants described more explicit forms of harassment, including verbal, physical, and digital misconduct. These included inappropriate touching, sexually explicit remarks, intrusive questions about private life, unsolicited digital contact, and unwanted advances by supervisors. Students also described situations in which initially professional interactions—such as clinical procedures or teaching encounters—were subsequently reframed in a sexualized manner. Participants emphasized that such reinterpretations were particularly disturbing because they transformed clinically focused interactions into personally exposing situations.

**Table 1:** Gendered and sexualized boundary violations

Subcode	Content	Quote
Academic/Professional Discrimination Based on Gender	Denial of teaching, preference for men, disadvantage for women, verbal or structural discrimination due to potential or existing motherhood, lack of support, discouragement from certain specialties	*“The doctor was standing at the operating table and […] refused to let her come to the table because she was a woman*,* because she was female.”* (P6) *“Are you sure you want to go into surgery as a woman?”* (P3)
Degrading Treatment Based on Gender	Infantilization (pet names, use of first-name), disrespectful treatment, insulting or humiliating comments	*„I’ve been called things like darling*,* bunny or little mouse by surgeons or other senior physicians.”* (P12)
Reproduction of Gender Stereotypes	Emphasis on and evaluation of gender roles, stereotypical comments, gender-related jokes	*“You see*,* surgery is as simple as cooking. But of course*,* you’re not supposed to say that to women these days. You always have to be careful that no one feels offended.”* (P5)
Objectification	Portrayal as objects, gender-related comments, reduction to gender or appearance	*“Today*,* I have two pretty girls accompanying me in the operating room.”* (P5)
Misinterpretation of Professional Role due to Gender	Addressing female doctors/students as nurses, communicating with the male instead of the female person	*“Even during my nursing internship*,* I was already referred to as Doctor*,* while women can be fully qualified physicians with a doctoral title and still be addressed as ‘nurse.’”* (P4)
Physical Harassment	Inappropriate touching, “seemingly accidental” touching	*„In the emergency department*,* a young resident came up behind me and placed both of his hands on my hips while I was inserting an IV line.”* (P12)
Direct Verbal Harassment	Sexually explicit or offensive statements, inappropriate questions or comments about private life	*“Right on the very first day he asked me how I use contraception and what matters to me about it. He also asked whether I have a boyfriend or how things are going in my private life.”* (P7)
Digital Harassment	Harassment through phone calls, messages, on social media	*“The message on Facebook was very intrusive to my privacy because it wasn’t limited to the work situation.”* (P8)
Unwelcome Advances	Invitations to private meetings, asking for phone numbers, intrusive behavior	*“He suggested that I could come to his office sometime to have a coffee.”* (P12)
Sexualization	Sexualized reinterpretation of clinical or teaching situations	*“He [the resident] stood behind me to help me find the right position. I didn’t perceive it as uncomfortable at the time because we were focused on the intubation. But then*,* another colleague of the doctor came along and mockingly said*,* ‘Oh*,* what are you doing? That looks naughty.’”* (P5) *“When I started with the local anesthesia*,* he kept saying things like whether I enjoy causing men pain in my free time.”* (P7)
Stage of Medical Training and Learning Environment	Nursing internship, clerkship, final year, voluntary social year, examination course, bedside teaching, operating room, ward round, clinical work with patients, theoretical teaching	*“In the operating room*,* there was definitely a rather sexist atmosphere.”* (P9)
Perpetrators and Affected Individuals	Patients, senior physicians, consultants, residents, fellow students	“*When the other student was tired*,* he asked her if she had slept too much with her boyfriend over the weekend.”* (P9)

These incidents occurred across all stages of medical training and were embedded in routine educational settings, including ward rounds, operating rooms, and classroom teaching. Both female and male students identified patients as perpetrators, while harassment by physicians was reported exclusively by female participants and most frequently involved residents or senior physicians. Students described both their own experiences and observations of others being subjected to harassment or discrimination.

### Theme 2: role insecurity & interpretive ambivalence

Participants reflected on how such situations aligned or conflicted with their understanding of themselves as future physicians (Table 2). A central element of these reflections was uncertainty and interpretive ambivalence. Students often questioned whether a boundary had truly been crossed, whether intent had been misread, or whether they were “*overreacting*”. Ambiguity regarding perpetrators’ motives, particularly in patient interactions, and the normalization of certain behaviors within clinical culture complicated the interpretation of incidents as harassment.

**Table 2:** Role insecurity & interpretive ambivalence

Subcode	Content	Quote
Perceived Affectedness	Assessment of own affectedness, relief at not being severely affected, guilt that others might be affected	*“When you presented the study during our final-year teaching session*,* I initially thought*,* ‘Hmm*,* it doesn’t really concern me.’ […] At first*,* I wouldn’t have described myself as a victim. But then I reconsidered the situation.“* (P5) “*I gradually became aware that these were boundary violations I didn’t have to accept.”* (P3).
Questioning Misinter-pretation of the behavior	Individuality of boundaries, sensitization, uncertainty in assessing the incident, gray area, lack of self-confidence, recognizing the perpetrator’s intent	*“Maybe it was really just meant nicely*,* and I’m simply misinterpreting it.” (P6).* *“Some of them were demented*,* so sometimes I couldn’t really say for sure whether it was an intentional act […] or due to dementia.” (P3)* *“I find it really not so easy at first to understand when someone in the hospital context crosses boundaries.”* (P7)
Undervalued Professional Role	Perception in the professional role, feeling not being taken seriously, position within the treatment team	*“I had the feeling that I was not seen as a doctor*,* but simply as a woman.” (P1)* *“I think we often project a certain sense of helplessness - at least I do.” (P11)*
Wish to fit in	Perception of the person, conflict avoidance, need for harmony, feeling the need to explain oneself, putting own needs aside	*“It was the first time I operated with him*,* and then to immediately stand out as someone who is seen as difficult - you do think about that.”* (P12) *“That general feeling of somehow being less valued […] and of easily being seen as the uptight or uncool person if you don’t laugh along.”* (P11)
Perspective-Taking and Empathy	Pity for the perpetrator, sympathy, reflection on consequences for others, separation of behavior from the person	*“Poor guy! Sure*,* it’s inappropriate*,* but somehow*,* I also felt sorry for him.”* (P3) *“You don’t want them to feel bad if I point out a boundary violation.”* (P3)
Professionalism	Responsibility for patients, understanding of professional role	*“Because you are still in this physician role […] you have to continue working with the patient.”* (P3) “*I think in the hospital*,* when dealing with patients*,* a certain level of professionalism is expected*,* and you may not want to step away from that*.” (P11)

Several participants also found it difficult to identify themselves as “*victims*”, especially when incidents did not involve physical contact. Experiences were frequently downplayed, and some students expressed relief at not having encountered more severe forms of abuse. At the same time, participation in the study itself prompted some to reconsider previously normalized experiences.

Students also described tensions between professional expectations and personal boundaries. Many felt pressure to appear resilient and “*not overly sensitive*”, particularly within hierarchical clinical environments. Some students felt harassment or discrimination undermined their role within the team. A strong desire to fit into existing social and professional structures contributed to hesitation in reacting to inappropriate behavior.

Students feared being perceived as overly sensitive (*“uptight”*,* “uncool”*) or disruptive (*“rebellious”*,* “a killjoy”*) if they challenged such conduct.

In patient interactions, empathy and professional responsibility further complicated interpretation. Some students felt reluctant to confront vulnerable patients, while others worried that assertive reactions might appear unprofessional or disrupt the therapeutic relationship. Harassment by supervisors or senior physicians was experienced as particularly challenging, as it undermined students’ professional legitimacy and sense of belonging within the clinical team. Overall, experiences of SH intersected with role insecurity inherent in early professional development.

### Theme 3: hierarchical dependency & cultural normalization

Students’ experiences of SH and GBD were embedded within hierarchical structures that shaped both perception and response (Table 3). Beyond formal hierarchy, participants described a broader cultural normalization of sexist remarks and gendered boundary violations within hospital environments. Such behaviors were often ignored by bystanders or reframed as harmless jokes, particularly in hierarchical specialties such as surgery. Some students reported adapting to these norms over time, gradually shifting their own tolerance thresholds and downplaying incidents to avoid being perceived as overly sensitive.

**Table 3:** Hierarchical dependency & cultural normalization

Subcode	Content	Quote
Social Norms	Societal perception of the issue	“*I think one reason I didn’t say anything was because I knew from others that it had happened to them as well and they hadn’t done anything either.”* (P8)
Organizational Culture	Normalization of experiences by the environment, shifting of boundaries, adaptation to hospital culture, male-dominated environment	*“That is just how it is. He is a surgeon. That’s just the type.”* (P9) *“Afterwards*,* a female resident told me*,* ‘You shouldn’t be surprised. He’s the only one I would allow to address me that way.’”* (P12)
Hierarchies and Power Structures	Intimidation, aggressiveness of perpetrators, perceived helplessness	*“You can’t do that. He’s simply a big shot here.”* (P12) “*Who am I to say anything?”* (P9)
Dependency Dynamics in the Educational Context	Dependency of learners, disadvantages for learning success in case of refusal, reprisals in examinations	*“After I said that I wasn’t allowed to go into the operating room at all for a week.” (P7)* *“He’s the examiner for the state exam… That’s a real powerful position.”* (P9)
Interactions and Reactions of Perpetrators	Interpretation of the perpetrator’s emotions, consequences following boundary setting, discrimination after rejection, unsuccessful attempts at setting boundaries.	*“[I] was no longer allowed to join [the perpetrator] in the operating room or accompany him to the patients.”* (P7)
Structural Constraints	Workload, lack of contact points/support structures, mistrust in the system, unfamiliar setting during internship, lack of social connection	*“It’s not that doors are closed to the topic*,* but there’s simply no capacity.”* (P2) *“I honestly wouldn’t know of a single hospital I’ve been to where I would know whom to turn to in a situation like that.”* (P8) *“I could have told a nurse*,* but they probably couldn’t have done much anyway.” (P8).*

As learners, students depended on supervisors for teaching opportunities, evaluations, and future career prospects. This dependency created structural vulnerability that limited their willingness to confront or report inappropriate behavior, particularly when perpetrators held positions of authority. Fear of academic repercussions was grounded in concrete experiences, as some participants described being excluded from learning opportunities after attempting to set boundaries.

Institutional conditions further limited responses. High workloads, lack of support, and unclear reporting structures reduced the likelihood that incidents would be addressed, and many students were unaware of available reporting pathways or doubted their effectiveness.

### Theme 4: adaptive silence & constrained agency

Students described strong emotional reactions to SH and GBD, including anger, fear, disgust, shame, and feelings of helplessness or insecurity (Table 4). While many participants reported developing emotional distance over time, some described persistent discomfort following such incidents.

**Table 4:** Adaptive silence & constrained agency

Subcode	Content	Quote
Emotional Reactions	Anger, resentment, disgust, fear, shame, powerlessness, sense of exposure, discomfort, helplessness, shock, surprise, overwhelm, tolerance	“*Now I […] feel uncomfortable for the next few hours or the rest of the day.”* (P2)
Reactive Protection Mechanisms	Freezing, silence, ignoring, continuing as if nothing happened, smiling/laughing, emotional distance	*“I was completely defenseless*,* totally startled*,* and couldn’t say anything.”* (P12) *“You’re overwhelmed in the situation*,* and then you often think you can just smile it off*,* swallow it*,* and carry on.”* (P5)
Distancing and Boundary-Setting Strategies	Leaving the situation, verbally setting boundaries, solidarity with others in the room, avoiding people or places	*“And then I […] said*,* ‘I’m leaving now*,*’ and then I left the room.”* (P7)
Critical Evaluation of Lack of Behavioral Reaction	Anger about own behavior, self-blame, disappointment, desire for self-assertion, expectation of own reaction, ambivalence	“*I thought it was disgusting*,* and I was angry with myself that I did not say anything but just ignored it.”* (P11) “*It’s actually embarrassing that one doesn’t say anything in a situation like that.”* (P12)
Rationalization, Minimization, and Suppression of the Experience	Downplaying, justification, resignation, acceptance of the situation, not using reporting mechanisms	*“Well*,* it wasn’t that bad*,* I wasn’t touched or anything.”* (P2) *“I just didn’t want to deal with it.”* (P11) *“You think about it back and forth in your head and then tell yourself*,* ‘It wasn’t that bad.’”* (P12).
Lack of Communication Skills as Barrier to Action	Lack of knowledge, lack of preparation or communication strategies	*“[…] because I wouldn’t have known an appropriate reaction that quickly.”* (P8)

Students’ responses were often characterized by passive, instinctive reactions aimed at avoiding further distress. Many reported freezing, remaining silent, or continuing the interaction as if nothing had occurred, while active confrontation was described as rare and particularly difficult when perpetrators held supervisory roles. Some students attempted to set verbal boundaries or distance themselves from the situation, but such responses were sometimes associated with negative consequences, including exclusion from learning opportunities or altered professional relationships. Post-incident strategies involved avoidance, such as staying away from perpetrators or locations.

Participants frequently reflected critically on their own reactions, expressing frustration, shame or self-blame for not having responded more assertively. At the same time, many described lacking strategies or training to respond appropriately in such situations. To cope with these experiences, some students minimized or rationalized incidents as “*not that bad*” or deliberately avoided reflecting on them in order to reduce emotional burden.

### Theme 5: institutional support & skills for boundary-setting

When reflecting on prevention and support, students articulated a clear need for both structural safeguards and practical competencies that would enable them to set professional boundaries more confidently (Table 5).

**Table 5:** Organizational strategies & skills for boundary-setting

Subcode	Content	Quote
Institutional Support	Low-threshold services, discussion with near peers, transparent processes with clear consequences	*“I think I would feel safer if it were clearer to me whom I can approach to report incidents.”* (P5) *“Who can I talk to? There should be trusted persons during medical school or the nursing internship.”* (P3).
Processing through Informal Networks	Talking to trusted peers, exchange with friends	*“I talked to a friend*,* and then I just forgot about it.”* (P11)
Cultural Change	Codes of conduct, reduction of hierarchies, role models, zero-tolerance policy, inclusive work environment, solidarity	*“I think it’s important*,* especially as a man*,* to speak up against it.”* (P4) *“It’s just not talked about much in medicine. Privately*,* yes*,* but not here.”* (P4).
Need for behavioral competence	Sensitization, communication training, desire for more quick-wittedness	*“I always thought I needed a sentence ready that I could say in that moment.”* (P12) “*I really wish I could be more quick-witted and able to stand up for myself.”* (P2).

Participants emphasized the importance of clear reporting structures, accessible and trusted contact persons, and transparent procedures with visible consequences. At the same time, many students were uncertain about existing support structures or doubted their effectiveness. They described reliance on informal peer networks for validation and emotional support. Students also described medical culture as one in which experiences of harassment are rarely discussed openly, contributing to a sense of isolation. They called for stronger institutional commitment at all hierarchical levels, including clear behavioral standards, inclusive communication, and visible leadership support to signal intolerance toward boundary violations. In addition to structural measures, participants emphasized the need for practical action competence. They highlighted the value of awareness training and communication strategies that would help them recognize harassment and respond more confidently in such situations or address incidents retrospectively.

**Fig. 1:**
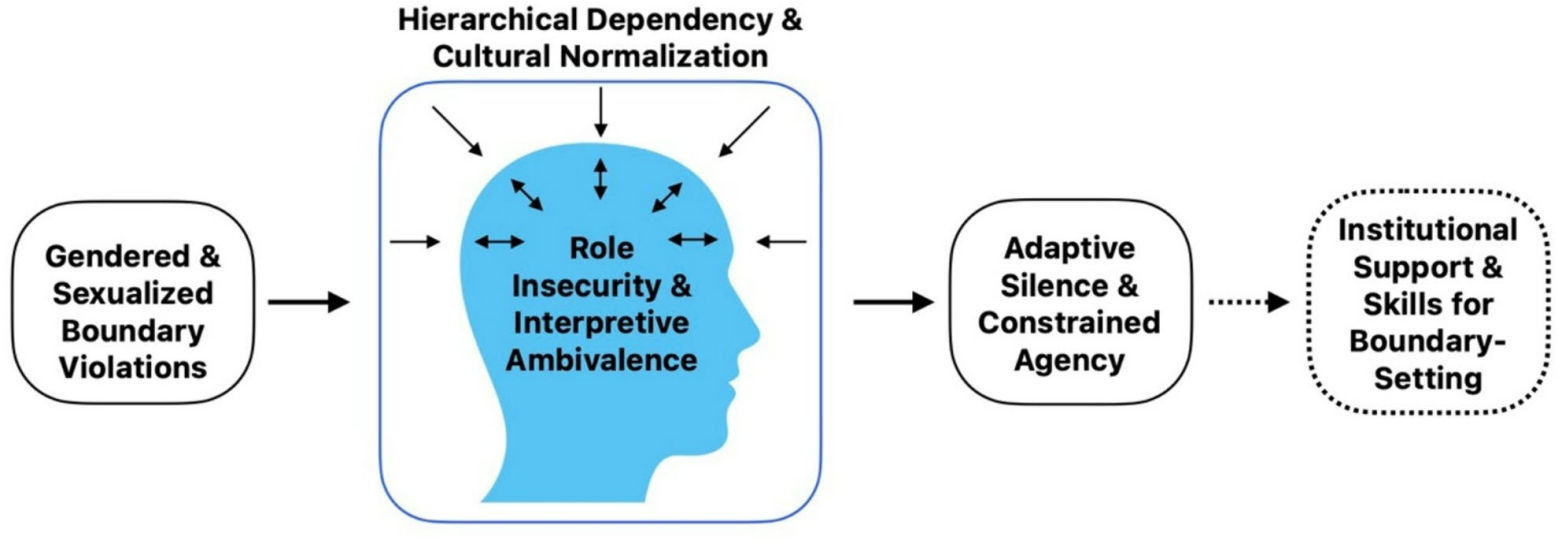
Conceptual model of students’ perceptions of and responses to gender-based discrimination and sexual harassment in medical training

Together, the five themes illustrate how experiences of boundary violations intersect with role insecurity, interpretive ambivalence, cultural normalization and hierarchical dependency, shaping students’ responses and contributing to adaptive silence and constrained agency, while also revealing the need for institutional support and practical response strategies (Fig. 1).

## Discussion

In this qualitative study we investigate the experiences of SH in medical education from the perspective of students. The findings align with existing literature on SH in workplace and academic settings [6–8, 23, 24, 34–37], but also highlight specific contextual factors relevant to medical education - including the therapeutic relationship with patients, students’ dependence on supervisors for learning opportunities and assessment, and the routine requirement for close physical contact during clinical skills training.

Our findings suggest that sexual harassment in medical education occurs within hierarchical training systems in which students depend on supervisors for access to learning opportunities and evaluation. At the same time, students are still developing their professional identities and learning how to navigate professional boundaries. This combination of structural dependency and role insecurity often limited students’ willingness or ability to confront misconduct, resulting in constrained agency and adaptive silence.

SH encompasses a wide range of behaviors and is closely related to, though not identical with, GBD. Although the opening question focused on SH, students frequently reported experiences of GBD. Their reports of harassment involved both patients (male and female) and faculty (all male), and the described forms aligned with categories from existing literature [23, 24, 37, 38]. In the context of practical training, harassment or discrimination by patients or instructors is a well-described problem, but is often unreported [6, 39]. Medical students, especially those in their early training, may struggle to identify inappropriate behavior as harassment [6]. No SH by peers was reported, although peer harassment was a significant part of data from other studies in Germany [6, 8, 10]. A specific form of GBD, often reported by participants, was misrecognition of roles within the treatment team, such as patients addressing female doctors or students as nurses. This issue has been noted in the healthcare context [40], but has not yet received significant attention in the literature on GBD.

### How does sexual harassment affect medical students?

Students reported negative emotions such as isolation, helplessness, shame, and anger in response to SH. Dissatisfaction with their own reactions often intensified these feelings, creating a dual emotional burden. Prior research links SH to depression, substance use, and sleep or eating disorders [35, 36, 41–45], as well as reduced academic satisfaction, motivation, and performance [36, 46, 47]. Consistently, participants described limited learning opportunities and academic disadvantages resulting from SH/GBD.

Experiences of SH and GBD intersected with inherent role insecurity in undergraduate medical training, amplifying vulnerability. Beyond describing emotional reactions, participants’ narratives revealed how experiences of SH and GBD were deeply intertwined with the development of their professional identity. Students did not only describe anger, fear, shame, or disgust; they reflected on their own reactions and questioned whether they had behaved in a sufficiently professional or assertive manner.

Harassment disrupted the professional relationship with patients, causing emotional strain and potentially affecting both students’ mental health and the quality of care [13, 39, 41]. Such behavior may include suggestive remarks, inappropriate touching, or the sexualization of medical procedures. These experiences can impair concentration and professional performance [34, 36, 41, 47] and may have long-term consequences for students’ sense of professional identity, trust in clinical relationships, and personal safety [13, 35, 47].

Participants in our study avoided specific people or situations to prevent further incidents, though this rarely provided relief. Some described internal conflict, fearing that avoidance might expose peers to harassment, and several reconsidered particular specialties, hospitals, or work environments for their future careers. In line with this finding, previous studies reported that affected students often protect themselves by avoiding certain people, locations, or events, skipping classes, or even altering their study or career plans [34, 36].

### How do medical students respond to boundary-crossing behavior?

Within this context of role insecurity and hierarchical dependency, students’ responses were often characterized by silence, ignoring the behavior, freezing, smiling, or rationalization; and linked to the feeling of helplessness and an inability to respond. This response, known as tonic immobility, is often observed in victims of sexual assault [48, 49]. Rather than reflecting individual weakness, these reactions emerged as adaptive strategies to preserve professional relationships and educational opportunities within a context perceived as unsafe for open resistance.

Ambiguity regarding intent, empathy toward patients, and uncertainty about appropriate professional conduct further complicated students’ responses. In response, students mostly approached peers and activated informal networks. Informal peer support provided emotional relief but rarely translated into formal reporting. Without visible institutional backing and clear role expectations, students may learn to adapt to boundary violations rather than to challenge them, which has implications for the normalization of misconduct in clinical culture.

### What factors influence students’ experiences and responses?

Consistent with prior research on SH in healthcare, students in this study struggled to recognize boundary violations, particularly when such behavior was normalized within the medical context [50]. Early in their training, many failed to identify inappropriate conduct and reported gradual desensitization to boundary crossings over time. Participants reported that their young age and lack of self-confidence influenced both their experiences and their responses. As younger students are particularly vulnerable to SH [13, 51], early interventions that raise awareness and strengthen institutional protection are essential, especially since some clinical placements are not formally integrated into university oversight.

Students perceived situations in which interactions initially understood as purely professional (such as drawing blood, performing physical examinations, or practicing clinical skills involving close physical contact) were subsequently reinterpreted or reframed in a sexualized way by patients or colleagues as particularly severe boundary violations.

Notably, responses differed depending on relational context. In interactions with patients, restraint was often justified by professional responsibility and empathy. Students felt obligated to continue care despite discomfort, particularly when patients were cognitively impaired or vulnerable. Students in this study reported difficulty responding to harassment by patients, as prioritizing patient care often led them to neglect their own well-being. Their dual role, as learners within a hierarchy and as responsible members of the care team, creates particular challenges. Students expressed a strong sense of responsibility toward their patients, leading them to tolerate boundary violations to maintain patient comfort or ensure continuity of care. This aligns with research showing that medical socialization fosters altruism and self-sacrifice [52], which may inhibit self-protection.

In interactions with supervisors, silence was more directly linked to fear of academic disadvantage or exclusion from learning opportunities. The hospital environment, characterized by rigid hierarchies and power dynamics, fosters a culture where inappropriate behavior is often normalized or overlooked [53], particularly in hierarchically structured specialties. Consistent with prior reports, this normalization discouraged students from speaking out, as nonconformity may have negative consequences for their academic and career progression [54]. In clinical training, especially in internships, learners rely on the willingness of supervisors to offer them learning opportunities. The asymmetric relationship between students and supervisors reinforces this dependency, making it difficult to address misconduct [53, 54]. Hierarchical dependency and cultural normalization operate as mutually reinforcing mechanisms. Within such an environment, challenging misconduct may threaten not only immediate relationships but also one’s educational trajectory and sense of belonging within the profession. Furthermore, many participants described a strong desire to adhere to existing social norms and avoid conflict. This tendency toward compliance, reinforced by hierarchical structures and implicit expectations within the medical culture, made it difficult for them to set boundaries.

### What forms of support do final year medical students consider helpful?

Students emphasized the need to strengthen their competence in managing harassment, including greater awareness of personal and professional boundaries, increased self-confidence, and effective communication strategies. Although communication training is now part of many medical curricula, SH in clinical settings remains insufficiently addressed. Workshops for faculty and residents have proven valuable in promoting intervention skills [55, 56], yet comparable training for medical students is still scarce, despite promising pilot initiatives [9, 57]. Evidence from Chinese college students shows that a clear understanding of SH increases the likelihood of active responses [58], underscoring the need for clear definitions and structured education.

Beyond education, students highlighted the importance of institutional support, requesting accessible resources and a broader cultural shift in clinical environments. Most were unaware of formal reporting systems and relied instead on informal networks such as peers or junior faculty. Institutional tolerance of SH is a major risk factor for its persistence [45, 59]. Thus, preventive organizational structures and effective support mechanisms are essential [60]. Awareness campaigns alone appear insufficient if students are not empowered to use available resources [9]. The diversity of individual needs and usage of support structures [61] underscores the importance of tailoring interventions and support systems to students’ specific circumstances [60]. Although participants did not explicitly mention psychological barriers such as fear of disbelief or victim-blaming, these factors likely influence underreporting and warrant further study.

### Recommendations

The findings of this study suggest that prevention strategies should directly address the contextual dynamics identified in students’ narratives, particularly (1) uncertainty in recognizing boundary violations, (2) role insecurity, (3) hierarchical dependency and concerns about professional consequences, (4) role conflicts when experiencing SH/GBD by patients, and (5) preference for informal support. Behavioral prevention aims to strengthen individual competencies and promote empowerment, while structural prevention seeks to establish a safe and respectful learning environment [62]. Priority should be given to structural prevention, as sustainable change requires visible institutional commitment [62]. Table 6 summarizes potential prevention strategies that we conclude from the results of our study, integrating both behavioral and structural prevention measures.

**Table 6:** Recommendations and prevention strategies derived from study findings

Key Finding	Implication	Recommended Measures
Uncertainty in identifying boundary violations	Students lack clarity about what constitutes SH/GBD in clinical contexts	Workshops defining SH/GBD with concrete clinical examples; case-based discussions; clear communication of institutional definitions
Role insecurity and vulnerable professional identity	Limited confidence in setting boundaries	Explicit integration of professional identity formation into curricula; mentoring and reflective supervision formats
Hierarchical dependency and fear of repercussions	Students hesitate to report misconduct by supervisors or faculty	Confidential and independent reporting pathways; visible anti-retaliation policies; transparent investigation procedures; designated trusted contact persons
Harassment by patients complicated by role expectations	Students struggle to balance professionalism and boundary-setting	Training in communication and de-escalation strategies; explicit guidance on managing inappropriate patient behavior; structured supervisory support during clinical placements
Preference for low-threshold support	Students turn to peers rather than institutions	Structured peer-support programs; accessible informal advisory spaces, low-threshold reporting (e.g. via online-chat, anonymous), information on available counseling and reporting structures

Because harassment by colleagues or supervisors is shaped by power asymmetries and fear of academic repercussions, institutions must ensure confidential and independent reporting pathways, transparent procedures, protection against retaliation, and consistent enforcement of codes of conduct [60, 63], to reduce the perceived risks of reporting. In contrast, harassment by patients occurs within the clinical encounter and is complicated by students’ role expectations and concerns about jeopardizing the therapeutic relationship. Educational interventions should therefore explicitly address how to manage inappropriate patient behavior while maintaining professionalism, including communication and de-escalation strategies, explicit guidance on managing inappropriate patient behavior, and real-time supervisory support. Across both contexts, our findings highlight students’ uncertainty in identifying boundary violations. Workshops that explicitly define SH and GBD, provide concrete examples from clinical settings, and inform students about available counseling and reporting structures may strengthen students’ ability to recognize and address misconduct [57]. Furthermore, PIF should be explicitly integrated into medical education, as role insecurity and dependency emerged as central themes in students’ experiences. Previous research shows that experiences of harassment and discrimination during clinical training can influence how students interpret professional norms and respond to professionalism dilemmas [64]. PIF describes the process through which learners internalize professional values, norms, and expectations and develop a sense of themselves as physicians [25, 65]. Supporting this process may strengthen students’ professional self-concept and help them set boundaries more confidently when confronted with inappropriate behavior. Finally, given participants’ preference for low-threshold support, structured peer-support initiatives should also be implemented to provide accessible spaces for exchange, reflection and mutual support [66, 67].

### Limitations

Despite careful planning, this study has several limitations. First, participation required willingness to disclose and reflect on experiences of SH or GBD in an interview context. Students who chose to participate may therefore represent individuals who had already recognized and cognitively processed such experiences. Consequently, the findings may not fully capture perspectives of students who would not label certain behaviors as harassment, who normalize boundary violations, or who avoid engaging with the topic altogether. This potential self-selection bias may have influenced the prominence of interpretive ambivalence and reflective identity work observed in the analysis. Second, the dual role of the interviewer may have introduced bias; however, objectivity was supported through the use of a structured interview guide, transparent coding procedures, and reflexive discussions during analysis. Third, female students were overrepresented in the sample, which may have influenced the thematic emphasis of the findings. Despite targeted recruitment efforts, comparatively few male students participated, which may reflect gender differences in identifying with or responding to the topic of sexual harassment. Previous research suggests that women are more likely than men to label certain behaviors as SH [68], which may partly explain the lower engagement of male students. In addition, the gender of the interviewer may have influenced willingness to participate or disclose experiences, as female students may have felt more comfortable discussing sensitive experiences with a female interviewer. At the same time, the gender distribution in the sample reflects the broader demographic trend of increasing female representation in German medical schools. Nevertheless, male perspectives - including differences in the perception, interpretation, and response to harassment, as well as potential differences in support needs - may be underrepresented. Gender differences in the perception, labeling, and reporting of sexual harassment have been described in the literature, with male victimization remaining comparatively understudied and potentially underreported. Fourth, the relative homogeneity in cultural and religious backgrounds restricts the diversity of experiences represented. Consequently, the findings cannot be generalized to all medical students but provide valuable qualitative insights into how harassment is perceived and experienced within a relatively homogeneous cohort. Future research should include more diverse and intersectional samples to explore how ethnicity, sexual orientation, gender identity, and cultural background shape experiences of harassment. Examining the perspectives of perpetrators and the long-term effects on students’ mental health, professional identity formation, and career trajectories would further deepen understanding of this complex issue.

## Conclusion

Our findings suggest that SH in medical education is experienced not merely as misconduct, but as a threat to emerging professional identity within a hierarchical training system. Harassment perpetrated by colleagues or supervisors is shaped by hierarchical relationships, power imbalances, and fears of academic or career-related repercussions, which may inhibit recognition and reporting. In contrast, harassment from patients occurs within the clinical encounter and is often complicated by students’ concerns about maintaining the therapeutic relationship and fulfilling professional role expectations. A key finding of this study is students’ uncertainty in identifying boundary violations within the medical context, underscoring the need for clearer definitions and concrete examples of inappropriate behavior in medical training. Young professionals in dependent roles face difficulties in resisting or reporting misconduct due to their vulnerable position within hierarchical structures. SH and GBD in medical education are not merely individual issues but reflect broader institutional and societal dynamics. Effective prevention therefore requires a multi-level approach combining education, institutional accountability, and cultural change to create safe and respectful learning environments.

## Supplementary Information

Supplementary Material 1: Definition, Interview-Guide and Questionnaire (Translation).

## Data Availability

The data that support the findings of this study are not openly available due to reasons of sensitivity and are available from the corresponding author upon reasonable request.

## Abbreviations

GBD: Gender-Based Discrimination
SH: Sexual Harassment
UKA: University Hospital Augsburg (*Universitätsklinikum Augsburg*)
PIF: Professional Identity Formation

## References

[1] Federal Anti-Discrimination Agency. Sexual Harassment in the Workplace (Antidiskriminierungsstelle des Bundes. Sexuelle Belästigung am Arbeitsplatz. https://www.antidiskriminierungsstelle.de/DE/ueber-diskriminierung/lebensbereiche/arbeitsleben/sexuelle-belaestigung-am-arbeitsplatz/sexuelle-belaestigung-am-arbeitsplatz-node.html. Accessed 2 Jan 2025.

[2] SchröttleM, Meshkova K, Lehmann C. Umgang mit sexueller Belästigung am Arbeitsplatz – Lösungsstrategien und Maßnahmen zur Intervention. Studie im Auftrag der Antidiskriminierungsstelle des Bundes. 2nd ed. Antidiskriminierungsstelle des Bundes; 2019. https://www.antidiskriminierungsstelle.de/SharedDocs/downloads/DE/publikationen/Expertisen/umgang_mit_sexueller_belaestigung_am_arbeitsplatz_kurzfassung.pdf?__blob=publicationFile&v=12.

[3] Hsiao CJ, Akhavan NN, Singh Ospina N, Yagnik KJ, Neilan P, Hahn P, et al. Sexual harassment experiences across the academic medicine hierarchy. Cureus. 2021. 10.7759/cureus.13508. 10.7759/cureus.13508 PMC7992916 33786217

[4] Fnais N, Soobiah C, Chen MH, Lillie E, Perrier L, Tashkhandi M, et al. Harassment and discrimination in medical training: a systematic review and meta-analysis. Acad Med. 2014;89:817–27. 10.1097/ACM.0000000000000200. 24667512 10.1097/ACM.0000000000000200

[5] Dzau VJ, Johnson PA. Ending sexual harassment in academic medicine. N Engl J Med. 2018;379:1589–91. 10.1056/NEJMp1809846. 30207831 10.1056/NEJMp1809846

[6] Schoenefeld E, Marschall B, Paul B, Ahrens H, Sensmeier J, Coles J, et al. Medical education too: sexual harassment within the educational context of medicine – insights of undergraduates. BMC Med Educ. 2021;21. 10.1186/s12909-021-02497-y. 10.1186/s12909-021-02497-y PMC7852293 33526025

[7] Jendretzky K, Boll L, Steffens S, Paulmann V. Medical students’ experiences with sexual discrimination and perceptions of equal opportunity: a pilot study in Germany. BMC Med Educ. 2020;20:56. 10.1186/s12909-020-1952-9. 32087726 10.1186/s12909-020-1952-9 PMC7036258

[8] Ludwig S, Jenner S, Berger R, Tappert S, Kurmeyer C, Oertelt-Prigione S, et al. Perceptions of lecturers and students regarding discriminatory experiences and sexual harassment in academic medicine – results from a faculty-wide quantitative study. BMC Med Educ. 2024;24. 10.1186/s12909-024-05094-x. 10.1186/s12909-024-05094-x PMC11044556 38658938

[9] Buchhold B, Wille J, Stracke S, Lutze S. Prävalenz sexualisierter Belästigung in einem Krankenhaus der Maximalversorgung. MMW Fortschr Med. 2024;166:9–18. 10.1007/s15006-024-4421-2. 10.1007/s15006-024-4421-2 39653950

[10] Förstel M, Vogt M, Drossard S. Sexual harassment at German medical schools – a national cross-sectional study. BMC Med Educ. 2026. 10.1186/s12909-026-08890-9. 10.1186/s12909-026-08890-9 PMC13049711 41761159

[11] Clemens V, Kuchenbaur M, Richter C, Oertelt-Prigione S, Taubner S, Fegert JM. Sexual harassment in academic medicine in Germany. JAMA Netw Open. 2025;8:e2518237. 10.1001/jamanetworkopen.2025.18237. 40569598 10.1001/jamanetworkopen.2025.18237 PMC12203272

[12] Barbier JM, Carrard V, Schwarz J, Berney S, Clair C, Berney A. Exposure of medical students to sexism and sexual harassment and their association with mental health: a cross-sectional study at a Swiss medical school. BMJ Open. 2023;13. 10.1136/bmjopen-2022-069001. 10.1136/bmjopen-2022-069001 PMC10151891 37105707

[13] McClain T, Kammer-Kerwick M, Wood L, Temple JR, Busch-Armendariz N. Sexual harassment among medical students: prevalence, prediction, and correlated outcomes. Workplace Health Saf. 2021;69:257–67. 10.1177/2165079920969402. 33331247 10.1177/2165079920969402

[14] Frank E, Carrera JS, Stratton T, Bickel J, Nora LM. Experiences of belittlement and harassment and their correlates among medical students in the United States: longitudinal survey. BMJ. 2006;333:682. 10.1136/bmj.38924.722037.7C. 16956894 10.1136/bmj.38924.722037.7C PMC1584373

[15] Patton MQ. Qualitative research and evaluation methods. 3rd ed. Thousand Oaks, California: Sage Publications; 2002.

[16] Flick U. Sozialforschung: Methoden und Anwendungen. Reinbek bei Hamburg: Rowohlt Taschenbuch; 2009.

[17] Adler M, Vincent-Höper S, Vaupel C, Gregersen S, Schablon A, Nienhaus A. Sexual harassment by patients, clients, and residents: Investigating its prevalence, frequency and associations with impaired well-being among social and healthcare workers in Germany. Int J Environ Res Public Health. 2021;18. 10.3390/ijerph18105198. 10.3390/ijerph18105198 PMC8153261 34068346

[18] Russell HA, Fogarty CT, Mcdaniel SH, Naumburg E, Nofziger A, Rosenberg T, et al. Am I making more of it than I should? Reporting and responding to sexual harassment. Fam Med. 2021;53. 10.22454/FamMed.2021.808187. 10.22454/FamMed.2021.808187 34077959

[19] Jenner S, Djermester P, Prügl J, Kurmeyer C, Oertelt-Prigione S. Prevalence of sexual harassment in academic medicine. JAMA Intern Med. 2019;179:108. 10.1001/JAMAINTERNMED.2018.4859. 30285070 10.1001/jamainternmed.2018.4859 PMC6583418

[20] Mahurin HM, Garrett J, Notaro E, Pascoe V, Stevenson PA, DeNiro KL, et al. Sexual harassment from patient to medical student: a cross-sectional survey. BMC Med Educ. 2022;22. 10.1186/s12909-022-03914-6. 10.1186/s12909-022-03914-6 PMC9710121 36451194

[21] Hinze SW. Am I being oversensitive? Women’s experience of sexual harassment during medical training. Health. 2004;8:101–27. 10.1177/1363459304038799. 15018720 10.1177/1363459304038799

[22] Nora LM, McLaughlin MA, Fosson SE, Stratton TD, Murphy-Spencer A, Fincher R-ME, et al. Gender discrimination and sexual harassment in medical education: perspectives gained by a 14-school study. Acad Med. 2002;77:1226–34. 10.1097/00001888-200212000-00018. 12480632 10.1097/00001888-200212000-00018

[23] Witte FM, Stratton TD, Nora LM. Stories from the field: students’ descriptions of gender discrimination and sexual harassment during medical school. Acad Med. 2006;81:648–54. 10.1097/01.ACM.0000232421.04170.d2. 16799291 10.1097/01.ACM.0000232421.04170.d2

[24] Leskinen EA, Cortina LM. Dimensions of disrespect: mapping and measuring gender harassment in organizations. Psychol Women Q. 2014;38:107–23. 10.1177/0361684313496549.

[25] Cruess RL, Cruess SR, Boudreau JD, Snell L, Steinert Y. Reframing medical education to support professional identity formation. Acad Med. 2014;89:1446–51. 10.1097/ACM.0000000000000427. 25054423 10.1097/ACM.0000000000000427

[26] Cruess RL, Cruess SR, Boudreau JD, Snell L, Steinert Y. A schematic representation of the professional identity formation and socialization of medical students and residents. Acad Med. 2015;90:718–25. 10.1097/ACM.0000000000000700. 25785682 10.1097/ACM.0000000000000700

[27] Helfferich C. Interviewplanung und Intervieworganisation. Die Qualität qualitativer Daten. Wiesbaden: VS Verlag für Sozialwissenschaften; 2009. pp. 167–93. 10.1007/978-3-531-91858-7_6.

[28] Przyborski A, Wohlrab-Sahr M. Qualitative Sozialforschung. 5th edition. Berlin: De Gruyter; 2021. 10.1515/9783110710663.

[29] Kuckartz U, Rädiker S. Qualitative Inhaltsanalyse. Methoden, Praxis, Computerunterstützung. 5th ed. Weinheim: Beltz Juventa; 2022.

[30] Kuckartz U. Einführung in die computergestützte Analyse qualitativer Daten. Wiesbaden: VS Verlag für Sozialwissenschaften; 2007. 10.1007/978-3-531-90664-5.

[31] Campbell JL, Quincy C, Osserman J, Pedersen OK. Coding In-depth Semistructured Interviews. Sociol Methods Res. 2013;42:294–320. 10.1177/0049124113500475.

[32] Tong A, Sainsbury P, Craig J. Consolidated criteria for reporting qualitative research (COREQ): a 32-item checklist for interviews and focus groups. Int J Qual Health Care. 2007;19:349–57. 10.1093/intqhc/mzm042. 17872937 10.1093/intqhc/mzm042

[33] Mey G, Ruppel PS. Qualitative Forschung. Sozialpsychologie und Sozialtheorie. Wiesbaden: Springer Fachmedien Wiesbaden; 2018. pp. 205–44. 10.1007/978-3-531-19564-3_14.

[34] Hill C, Silva E. Drawing the line - sexual harassment on campus. Washington: American Association of University Women Educational Foundation; 2005.

[35] Tang AL, Seiden AM. Sexism and sexual harassment: considering the impact on medical students, residents, and junior faculty. Laryngoscope. 2018;128:1985–6. 10.1002/lary.27195. 30058159 10.1002/lary.27195

[36] Huerta M, Cortina LM, Pang JS, Torges CM, Magley VJ. Sex and power in the academy: modeling sexual harassment in the lives of college women. Pers Soc Psychol Bull. 2006;32:616–28. 10.1177/0146167205284281. 16702155 10.1177/0146167205284281

[37] Tameling J-F, Lohöfener M, Bereznai J, Tran TPA, Ritter M, Boos M. Extent and types of gender-based discrimination against female medical students and physicians at five university hospitals in Germany - results of an online survey. GMS J Med Educ. 2023;40:Doc66. 10.3205/zma001648. 38125897 10.3205/zma001648 PMC10728668

[38] Fitzgerald LF, Gelfand MJ, Drasgow F. Measuring sexual harassment: theoretical and psychometric advances. Basic Appl Soc Psych. 1995;17:425–45. 10.1207/s15324834basp1704_2.

[39] Liaw RDY, Ling DCT, Vuli LJ, Loch C, Adam LA. It’s just inappropriate: harassment of dental students by patients. J Dent Educ. 2022;86:605–14. 10.1002/jdd.12854. 34951016 10.1002/jdd.12854

[40] Hennein R, Poulin R, Gorman H, Lowe SR. Gender discrimination and mental health among health care workers: findings from a mixed methods study. J Womens Health. 2023;32:823–35. 10.1089/jwh.2022.0485. 10.1089/jwh.2022.0485 PMC10354310 37256783

[41] Mushtaq M, Sultana S, Imtiaz I. The trauma of sexual harassment and its mental health consequences among nurses. J Coll Physicians Surg Pak. 2015;25:675–9. 26374365

[42] McGinley M, Wolff JM, Rospenda KM, Liu L, Richman JA. Risk factors and outcomes of chronic sexual harassment during the transition to college: Examination of a two-part growth mixture model. Soc Sci Res. 2016;60:297–310. 10.1016/j.ssresearch.2016.04.002. 27712687 10.1016/j.ssresearch.2016.04.002 PMC5116326

[43] Vu M, Li J, Haardörfer R, Windle M, Berg CJ. Mental health and substance use among women and men at the intersections of identities and experiences of discrimination: Insights from the intersectionality framework. BMC Public Health. 2019;19. 10.1186/s12889-019-6430-0. 10.1186/s12889-019-6430-0 PMC6345035 30674293

[44] Blindow KJ, Cedstrand E, Elling DL, Hagland M, Bodin T. Gender-based violence and harassment at work and health and occupational outcomes. A systematic review of prospective studies. BMC Public Health. 2024;24:1788. 10.1186/s12889-024-19304-0. 38965519 10.1186/s12889-024-19304-0 PMC11225130

[45] Willness CR, Steel P, Lee K. A meta-analysis of the antecedents and consequences of workplace sexual harassment. Pers Psychol. 2007;60:127–62. 10.1111/j.1744-6570.2007.00067.x.

[46] Rosenthal M, Freyd J. Sexual violence on campus: no evidence that studies are biased due to self-selection. Dignity J Sex Exploit Violence. 2018;3. 10.23860/dignity.2018.03.01.07.

[47] Hill MR, Goicochea S, Merlo LJ. In their own words: stressors facing medical students in the millennial generation. Med Educ Online. 2018;23:1530558. 10.1080/10872981.2018.1530558. 30286698 10.1080/10872981.2018.1530558 PMC6179084

[48] Möller A, Söndergaard HP, Helström L. Tonic immobility during sexual assault – a common reaction predicting post-traumatic stress disorder and severe depression. Acta Obstet Gynecol Scand. 2017;96:932–8. 10.1111/aogs.13174. 28589545 10.1111/aogs.13174

[49] Kalaf J, Coutinho ESF, Vilete LMP, Luz MP, Berger W, Mendlowicz M, et al. Sexual trauma is more strongly associated with tonic immobility than other types of trauma – A population based study. J Affect Disord. 2017;215:71–6. 10.1016/j.jad.2017.03.009. 28319694 10.1016/j.jad.2017.03.009

[50] Hawes AM, Gondy K. Sexual Harassment in Medical Education: How We Can Do Better. J Gen Intern Med. 2021;36:3841–3. 10.1007/s11606-021-06960-w. 34145520 10.1007/s11606-021-06960-w PMC8642565

[51] Wood L, Hoefer S, Kammer-Kerwick M, Parra-Cardona JR, Busch-Armendariz N. Sexual harassment at institutions of higher education: prevalence, risk, and extent. J Interpers Violence. 2021;36:4520–44. 10.1177/0886260518791228. 30071790 10.1177/0886260518791228 PMC10676016

[52] Vaidyanathan B. Professional socialization in medicine. AMA J Ethics. 2015;17:164–70. 10.1001/virtualmentor.2015.17.02.msoc1-1502. 25676232 10.1001/virtualmentor.2015.17.02.msoc1-1502

[53] Chan ZC, Chien WT, Henderson S. Power dynamics in the student-teacher relationship in clinical settings. Nurse Educ Today. 2017;49:174–9. 10.1016/j.nedt.2016.11.026. 27984796 10.1016/j.nedt.2016.11.026

[54] Hayward L, Mott NM, McKean EL, Dossett LA. Survey of student mistreatment experienced during the core clinical clerkships. Am J Surg. 2023;226:13–8. 10.1016/j.amjsurg.2022.12.022. 36669940 10.1016/j.amjsurg.2022.12.022

[55] Goldenberg MN, Cyrus KD, Wilkins KM. ERASE: a new framework for faculty to manage patient mistreatment of trainees. Acad Psychiatry. 2019;43:396–9. 10.1007/s40596-018-1011-6. 30523539 10.1007/s40596-018-1011-6

[56] Hock LE, Barlow PB, Scruggs BA, Oetting TA, Martinez DA, Abràmoff MD, et al. Tools for responding to patient-initiated verbal sexual harassment: a workshop for trainees and faculty. MedEdPORTAL. 2021;17:11096. 10.15766/mep_2374-8265.11096. 33598539 10.15766/mep_2374-8265.11096 PMC7880260

[57] Drossard S, Warnken I. Teaching medical students to navigate workplace harassment – preliminary experiences from a pilot workshop in Germany. BMC Med Educ. 2025;25:1251. 10.1186/s12909-025-07853-w. 40931361 10.1186/s12909-025-07853-w PMC12421763

[58] Li X, Gu X, Ariyo T, Jiang Q. Understanding, experience, and response strategies to sexual harassment among chinese college students. J Interpers Violence. 2023;38:2337–59. 10.1177/08862605221101183. 35546082 10.1177/08862605221101183

[59] Johnson PA, Widnall SE, Benya FF, editors. Sexual harassment of women: climate, culture, and consequences in academic sciences, engineering, and Medicine. Washington, D.C.: National Academies; 2018. 10.17226/24994. 29894119

[60] Plener PL, Fegert JM, Wolff M. Konsequenzen für die Ausbildung. Hochschulen als riskante Orte und als Orte der Prävention. In: Fegert JM, Wolff M, editors. Kompendium „Sexueller Missbrauch in Institutionen. Entstehungsbedingungen, Prävention und Intervention. Weinheim: Beltz Juventa; 2015. p. 683–94.

[61] Bhattacharya A, Casey EA. Help-Seeking patterns among students experiencing sexual harassment: a latent class analysis. J Interpers Violence. 2024;39:3543–65. 10.1177/08862605241233269. 38415625 10.1177/08862605241233269

[62] Bundesportal. Sicherheit und Gesundheitsschutz am Arbeitsplatz. https://verwaltung.bund.de. Accessed 21 Oct 2025.

[63] Pantelmann H, Blackmore S, editors. Sexualisierte Belästigung, Diskriminierung und Gewalt im Hochschulkontext - Herausforderungen, Umgangsweisen und Prävention. Wiesbaden: Springer Fachmedien Wiesbaden; 2023. 10.1007/978-3-658-40467-3.

[64] Monrouxe LV, Rees CE, Endacott R, Ternan E. Even now it makes me angry’: health care students’ professionalism dilemma narratives. Med Educ. 2014;48:502–17. 10.1111/medu.12377. 24712935 10.1111/medu.12377

[65] Sternszus R, Steinert Y, Razack S, Boudreau JD, Snell L, Cruess RL. Being, becoming, and belonging: reconceptualizing professional identity formation in medicine. Front Med (Lausanne). 2024;11. 10.3389/fmed.2024.1438082. 10.3389/fmed.2024.1438082 PMC11383779 39257893

[66] Drossard S, Haertl A. Development and implementation of digital peer mentoring in small groups for first-year medical students. GMS J Med Educ. 2024;41(1):Doc11. 10.3205/zma001666. 38504864 10.3205/zma001666 PMC10946215

[67] Ye C, Xie Y, Pu S, Yao L. The influence of peer support on medical students’ professional psychological help-seeking intention: the chain mediating role of professional psychological help-seeking stigma and self-efficacy. BMC Medical Education. 2026. 2026. 10.1186/S12909-026-08802-X. 10.1186/s12909-026-08802-x PMC13041481 41723414

[68] Zhou Y, Nguyen H-HD, Revier MS, Krueger KR, Sackett PR. An updated examination of gender differences in sexual harassment perception: A meta-analysis and a survey study. J Occup Health Psychol. 2024;29:373–408. 10.1037/ocp0000391. 39699626 10.1037/ocp0000391

