# Supplementary material for: #MEDToo – sexual harassment in medical education: perceptions and coping strategies of medical students in Germany, a qualitative study
Source: BMC Med Educ. 2026 Apr 2;26:584. doi: 10.1186/s12909-026-09090-1 (PMC13064183; doi:10.1186/s12909-026-09090-1)
Supplement: Supplementary file 1 — Supplementary Material 1: Definition, Interview-Guide and Questionnaire (Translation). [file 12909_2026_9090_MOESM1_ESM.docx]

## Supplementary Material

Appendix 1: Definition, Interview-Guide and Questionnaire (Translation)

| **Introduction and Definition** |
| --- |
| In this interview, I would like to talk with you about your experiences with sexual harassment. According to German equality legislation, harassment occurs when an unwanted behavior is intended or has the effect of violating another person’s dignity - that is, when it insults, humiliates, or shames someone. Such behavior can be deliberate or unintentional. Sexual harassment refers to unwanted behavior that is sexualized or gender-related in nature. Examples include sexual innuendo or inappropriate physical contact.  Sexual harassment may constitute a criminal offense under certain circumstances. However, behaviors that are not (yet) legally punishable can still be experienced as inappropriate, intrusive, disrespectful, unpleasant, or degrading. Every person has an individual boundary for when a behavior constitutes a violation of their dignity, and these boundaries must be respected by everyone.  In the context of medical education, examples of sexual harassment may include:   - A patient asks a student inappropriate questions about their private or romantic life. - A patient comments on a student’s physical appearance (whether suggestively, condescendingly, or “intended as a compliment”). - A patient intentionally or “accidentally” touches a student on an intimate body part. - A lecturer stares at a student’s cleavage. - A patient makes a sexually suggestive comment or invites inappropriate touching. - A lecturer tells jokes with sexist content. - A patient comments on a student’s gender. - And many other possible examples. |
| **Questionnaire** |
| How old are you? |
| What gender do you identify with? |
| What is your nationality? |
| What is your religion? |
| Have you worked in a medically related field before starting your studies (e.g., nursing, emergency medical services, physiotherapy, etc.)? |
| If yes: How long did you work in patient care before beginning your medical studies? |
| Guiding Questions (Interview Guide) |
| During your medical studies, have you ever experienced sexual harassment? Could you tell me about it? |
| How did you perceive this situation? |
| How did you respond in that situation? |
| What do you think influenced your reaction? |
| How did you experience the reactions of other people who were present? |
| Have you encountered comparable situations outside of medical school, and did you respond differently there? |
| How would you assess your own competence in dealing with such situations? |
| How do you envision a learning environment in which you would feel safe? |
| Is there anything else you would like to add? |
